# Effects of Impurities and Ageing on the Functional and Rheological Properties of Asphalts with Additives from Recovered and Pyrolysis-Processed Plastics

**DOI:** 10.3390/ma17143451

**Published:** 2024-07-12

**Authors:** Marcin Daniel Gajewski, Renata Horodecka, Wojciech Bańkowski, Krzysztof Mirski, Aleksandra Grzegórska, Maciej Kłopociński

**Affiliations:** 1 Road and Bridge Research Institute, 03302 Warsaw, Poland; rhorodecka@ibdim.edu.pl (R.H.); wbankowski@ibdim.edu.pl (W.B.); kmirski@ibdim.edu.pl (K.M.); 2 Green Park VI Sp. z o.o., 87700 Aleksandrów Kujawski, Poland; a.grzegorska@greencapitalsa.com (A.G.); m.klopocinski@greencapitalsa.com (M.K.)

**Keywords:** plastics, recycling, asphalt, rheological properties, road pavement, ageing, impurities

## Abstract

This article is a continuation of work on the use of plastic waste (such as PP, PS, LDPE, HDPE, and their mixtures) processed in the proprietary pyrolysis process as asphalt additives. The article carried out detailed tests of the mixes of selected additives with pen-graded bitumen 50/70, taking into account, among others, the influence of impurities and the ratio of PE to PP in the additives as well as short- (RTFOT) and long-term (RTFOT + PAV) ageing. An extensive research program was carried out, including functional and rheological tests in a wide range of temperatures. First, tests of stability and adhesion to various types of aggregates were carried out, demonstrating the usefulness of the proposed additives. Then, the elastic recovery and the impact of technological ageing on penetration, Fraass breaking temperature, and plasticity range were assessed. The same binder mixes were subjected to rheological tests in a wide range of technological and operational temperatures, assessing, among others, viscosity, the norm of the complex shear modulus, elastic recovery and compliance in the MSCR test, and stiffness in the bending beam rheometer. This entire class of tests was carried out for clean samples and those containing impurities, indicating their impact on individual material parameters.

## 1. Introduction

The reason for the analyses undertaken into the possibility of using recycled plastics as additives to asphalts was the positive results presented, among others, in the papers [1,2,3,4,5], where the development of this concept from the modification of road asphalts through the replacement of binder by recycled plastics to the making of modular road components entirely from plastics was presented. Unfortunately, a broader view of the issue dampens the optimism somewhat and calls for the question of whether the addition of plastics in road pavements will lead to the production of excessive amounts of microplastics generated by road pavement abrasion. This question is answered by the authors of the paper [6], concluding that this issue should be verified experimentally, e.g., in a DSD study [7]. The use of this type of modification has a beneficial effect on the environment, but it is quite difficult to quantify. An attempt to determine the benefits of using PE pyrolytic waxes was made in a paper [8] estimating a reduction in environmental costs of up to 12%. In the case of the approach analysed in this paper, not only post-pyrolysis oils but also the solid fraction are used to enrich road asphalts, so it is to be expected that the environmental benefits may be greater.

The results presented in the work [9], where a comparative study was carried out for PE within RILEM, demonstrate how important the problem of the applicability of waste plastics is. This also demonstrates that we are gradually moving from basic research to standardisation of research verifying the thermal, chemical, and mechanical effects resulting from the use of plastics as additives and thus their more extensive application [10,11]. In general, recycled wastes are added to the binders or mixtures using the so-called wet or dry methods, respectively [12,13]. Recently, however, a new trend has emerged for using this type of waste processed into powder [14]. Plastic powders are used as agents for covering the surface of aggregates (including acidic ones [15]), improving the properties of the obtained mineral–asphalt mixtures (better adhesion, etc.) [16].

This paper is a continuation of the work [17] that carried out a detailed evaluation of the pyrolysis-processed [18,19] plastics on the properties of road asphalts. A detailed introduction on the use of plastics as additives in asphalt binders is therefore omitted here, along with a discussion of the current state of knowledge on the subject, which is included in this referenced paper (cf. the studies cited there and the review studies [20,21,22,23]). Although it has only been a few months since the publication of the aforementioned paper, there is a wealth of new pieces of literature on the recycling of plastics in asphalt pavements.

The aforementioned paper [17] presents the results of basic tests such as penetration (Pen25), PiK softening point ($T_{PIK}$), and Fraass breaking temperature ($T_{FRAASS}$) carried out on 56 mixtures of 50/70 and 70/100 road asphalts with pyrolytic additives made from plastics such as PP, PS, LDPE, HDPE, and their mixtures. In general, the samples prepared differed in the type of plastics added, their mass share, and the pyrolysis process parameters, i.e., temperature and processing time. A correlation analysis was carried out between these parameters and the properties of the modified asphalts. On the basis of the results obtained, using the author’s method of evaluation, those materials and material mixtures were identified that most favourably influence the functional properties of the base bitumen (50/70 and 70/100). Four additives in the so-called “soft asphalts” group (numbered 8, 20, 22, and 37) and five additives in the so-called “hard asphalts” group (numbered 3, 5, 7, 17, and 26) were identified. Among the additives selected for further analysis, additives numbered 37 and 26 deserve special attention, as they were produced by pyrolysis of mixed plastics (PE + PP + PET + PS). It is these two additives that will be the subject of the extended functional and rheological studies described in this paper.

The main goals of the paper are to analyse the influence of contamination of plastics subject to pyrolysis, their susceptibility to ageing, or the best percentage ratio of PP/PE on functional and rheological properties of obtained bitumen mixtures. Analysing the impact of the impurity content in the pyrolysed feedstock has a very practical dimension. From the point of view of possible practical applications of this technology, bringing plastic waste to a situation of perfect separation and perfect cleanliness requires a significant amount of work, materials from non-renewable sources (e.g., water), and energy, and sometimes it is not even possible. Suppose we prove that a small content of impurities does not have an excessively negative impact on the functional and rheological properties. In that case, we will make the potential application of the proposed approach more realistic and cost-effective (however, in this work we do not analyse the economic effectiveness of the proposed solution). Additionally, the work also considers the influence of changes in the proportions between the mass shares of the dominant plastics PE and PP as well as the effect of technological and operational ageing.

## 2. Selection of Additives for Extended Functional and Rheological Testing

**Figure 1:**
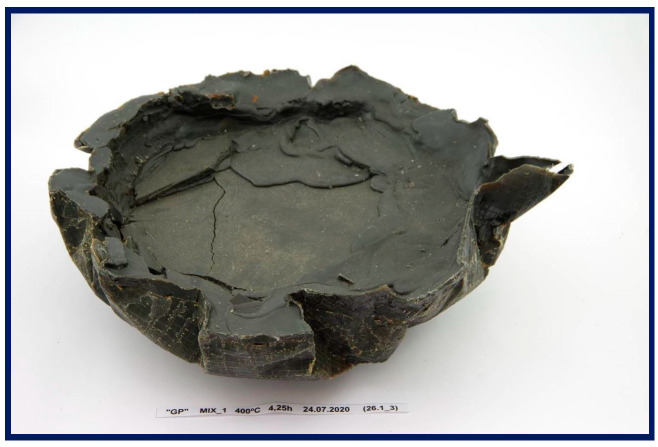
Additive number 26 (pyrolysis product).

**Figure 2:**
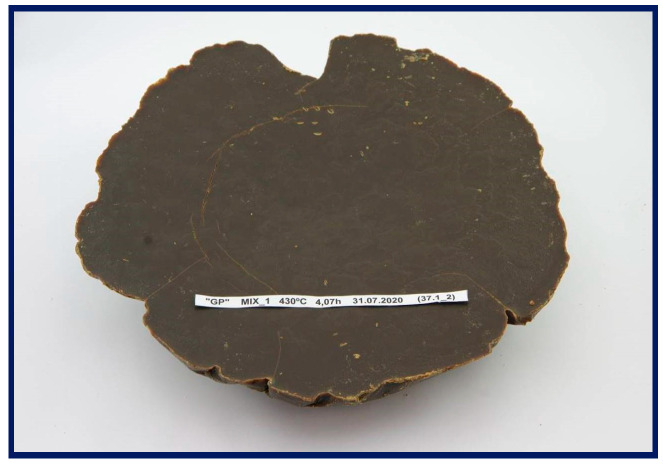
Additive number 37 (pyrolysis product).

For the testing of binders for functional and rheological characteristics, additives 26 and 37 (designated as GP MIX) were selected, which scored high in the analysis using the objective function (87 and 84, respectively, with a maximum value of 100, cf. [17]), while having the greatest practical potential. This is due to the fact that they consist of a mixture of plastics found in waste, which reduces the need for waste separation. The selected additives (26 and 37, cf. Figure 1 and Figure 2) were formed from the same waste mixture, but through different technological processes. They differed in the way in which they managed the hydrocarbon vapours (and the condensate obtained from the separation and condensation of the vapours) resulting from the process. Additive 26 was obtained by a process that produces a by-product in the form of a liquid, broad hydrocarbon fraction (condensate is not a component of the additive and is a by-product). Additive 37 was obtained by a process that does not produce a by-product in the form of a liquid, broad hydrocarbon fraction [19] (condensate is a component of the additive). The mass percentages of the individual plastics included in additives 26 and 37 are shown in Table 1. All the results presented hereafter were obtained on mixtures of 50/70 road asphalt with 5% (by weight) of either 26 or 37. The following blends (here, 26 and 37) were prepared as mixtures of pure binder and powder obtained from crushed pyrolysis products. The procedure and conditions leading to obtaining subsequent mixtures were presented in detail in [17].

The paper [17] also shows that the reproducibility of the test results for penetration, softening temperature, and stability of asphalt mixtures and re-mixed additives is at a very good level. The only problem was obtaining satisfactory reproducibility of the Fraass breaking temperature for additive 37, which was solved by more careful control of the waste treatment process.

A further extended analysis considered the possibility of contamination of additives at 0, 20, and 40%, and in the case of additive 26, variation in the mass proportions of polyethylene and polypropylene (48/52, 58/42, and 68/32, respectively) was taken into account. According to Table 1, the total mass share of PE and PP is 98%. Sample determinations are shown in Table 2.

For example, a 20% impurities share should be understood as follows:

- Materials other than PE, PP, PET, and PS are rejected from the unwashed municipal waste stream;
- A mixture with the specified mass proportions is prepared from the segregated materials;
- The mixture prepared in this way constitutes 80% by weight of the pyrolysis feedstock;
- The remaining 20% is a non-segregated mix of the typical municipal waste stream.

Impurities, therefore, include among others paper (cardboard), textiles, organic materials, metals, and other plastics (such as rubber, ABS). The inclusion of the proportion of impurities in the study has a very significant impact on the assessment of the feasibility of producing the analysed additives in a real technological process, where production sterility is difficult to control.

In addition, the effects of short-term (technological ageing) and long-term (operational ageing) ageing on the basic material parameters characterising additive mixtures with 50/70 road asphalt are considered further in this paper. Short-term ageing is simulated by the RTFOT process (in accordance with PN-EN 12607-1:2014-12), while operation-aged asphalt is subjected to RTFOT and PAV processes sequentially (cf. EN 14769:2012).

## 3. Evaluation of Stability, Elastic Recovery, and Adhesion to Aggregates, Taking into Account the Share of Impurities and the PE/PP Ratio

**Figure 3:**
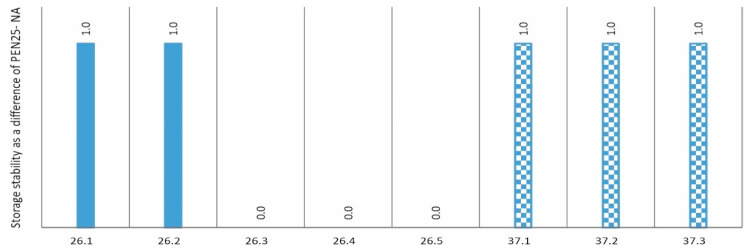
Stability—difference in Pen25 (non-aged condition—NA (not aged), values in penetration units—0.1 mm).

**Figure 4:**
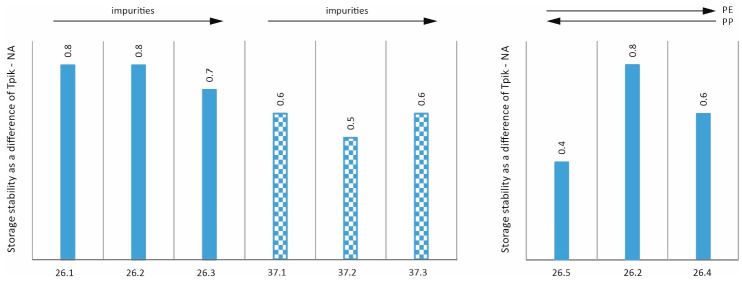
Stability—difference in $T_{PIK}$ (NA state, values in degrees Celsius).

A fundamental criterion for the suitability of an additive for asphalt is its stability, i.e., resistance to segregation at high temperatures [24,25,26]. Therefore, stability tests were carried out on mixtures of asphalt 50/70 with different versions of additives 26 and 37 in accordance with PN-EN 13399:2017-12. The method, known as the tube test, involves annealing the binder in a special tube and then determining the change in Pen25 and TPiK of the top and bottom of the sample (according to EN 1426:2015-08 and EN 1427:2015-08). Figure 3 shows the difference in Pen25 (top to bottom of sample) after the stability determination. Figure 4, on the other hand, shows the difference in softening temperature (top to bottom of sample) after the stability determination. All experimental results presented further are determined as the average value from three repetitions. Such an approach cannot be named a statistical one but it allows for the elimination of random errors.

Analysing the stability results in terms of penetration presented in Figure 3, it can be concluded that the stability is at a very good level and neither the degree of impurities nor the value of the PE/PP ratio affects it. Nevertheless, an analysis of the graph in Figure 4 allows initial conclusions to be drawn. It appears that an increasing proportion of impurities has a beneficial effect on the stability of the resulting mixture. Similar effects were observed in the paper [27] where the bitumen with additive of lignin containing waste showed good stability. The increase in the proportion of PE in the mixture also has a similar effect (cf. mixtures 26.1-3 of 26.4). It also follows that a stability test based on softening point values discriminates better between individual mixtures than a stability test based on penetration [28]. In fact, the problem of thermal or storage stability is very important in view of applying the plastics in the form of powders [29] or in other forms [30,31,32].

**Figure 5:**
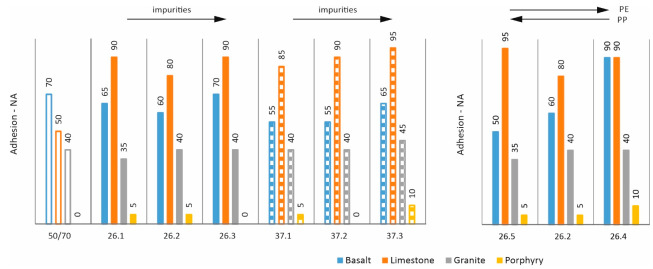
Adhesion to aggregates—(NA status, values in percent).

Another test that was carried out was to evaluate the adhesion of the analysed mixtures to basic road aggregates such as basalt, limestone, granite, and porphyry. The test was carried out using the cooking method according to PN-B-06714:84. Based on the results shown in Figure 5, it can be concluded that the additives affect the adhesion of the asphalts favourably in the case of limestone and porphyry. In the case of basalt, they slightly reduce adhesion and have little effect in the case of granite. As demonstrated in [17], even a small addition of an adhesive agent allows very good adhesion in all cases.

**Figure 6:**
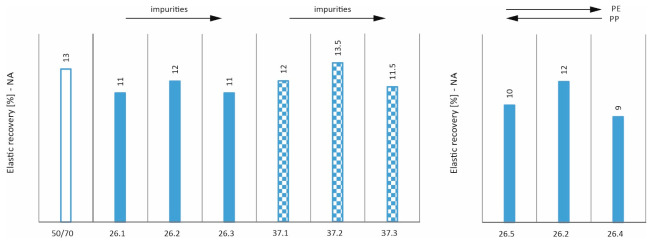
Elastic recovery (NA condition).

The introduction of additives into road asphalts aims to improve its physical and functional properties. By adding plastics, it is hoped that the resulting asphalt will have characteristics similar to polymer-modified asphalts (e.g., SBS) [33,34]. Therefore, an elastic recovery test of the prepared mixtures was carried out in accordance with EN 13398:2017-12. The results in Figure 6 show that the majority of the mixtures achieved the elastic recovery of more than 10%. This value is at a level typical of road asphalts and is far from the properties of polymer asphalts. It can also be seen that the introduction of impurities does not adversely affect the elastic recovery values obtained. Nevertheless, changing the PE/PP ratio leads to a reduction in the elastic recovery value, which would suggest that the 58/42 ratio chosen is the best.

## 4. Effect of Technological Ageing on Basic Functional Parameters

**Figure 7:**
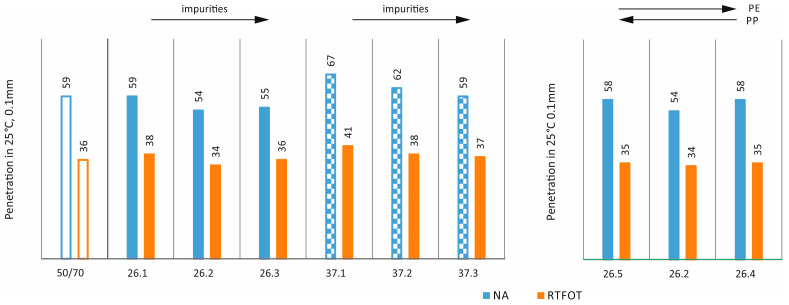
Effect of short-term ageing on penetration values.

**Figure 8:**
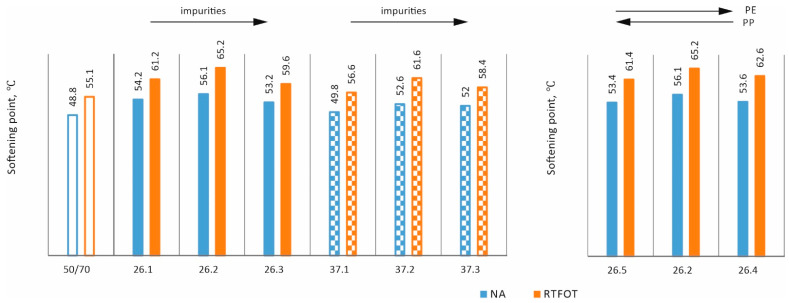
Effect of short-term ageing on softening point values.

**Figure 9:**
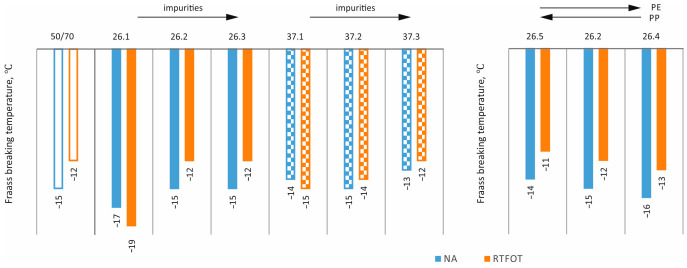
Effect of short-term ageing on Fraass breaking temperature values.

**Figure 10:**
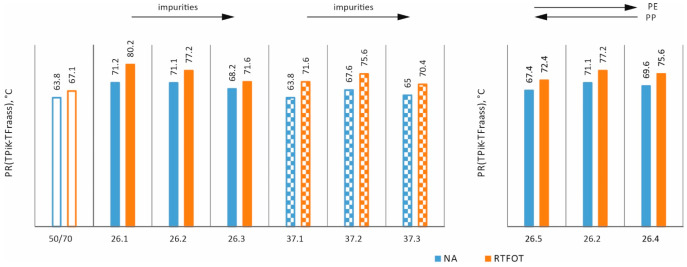
PR–plasticity range (NA state and after RTFOT ageing).

In order to assess the effects of ageing [35] and impurities on the properties of asphalts with processed plastic additives, the following tests were carried out: penetration at 25 °C, softening point, and Fraass breaking temperature on mixtures in the original state (NA) and after short-term ageing (RTFOT). The results obtained are shown as bar graphs in Figure 7, Figure 8, Figure 9 and Figure 10.

It can be seen from Figure 7 that the impurity content for both additives and both states leads to a decrease in penetration values. In the case of the PE/PP ratio, it can be concluded that both a reduction and a reduction in the proportion of PE in relation to the 58/42 ratio slightly increase the penetration value in the original state and have no major effect in the case of technological ageing. Figure 8 similarly shows the results obtained for the individual mixtures in the case of the softening temperature. In all cases, it can be seen that technological ageing increases the softening temperature values (a typical and expected effect). In addition, in all cases, the additives used increased the softening temperature compared to the base asphalt. The impact of the impurity content and PE/PP ratio for this study is not clear.

The situation is different for the Fraass breaking temperature. It is evident that additive 26 had a very favourable effect on this parameter (reducing the fracture temperature by 2 and 5 °C of the mixture in the original state and post-RTFOT, respectively). The addition of impurities neutralised this benefit, but did not worsen the parameter compared to the base asphalt. In contrast, no such significant improvement is apparent for additive 37, and the impurities content has little effect on the results obtained.

The parameter presented in Figure 10, called the plasticity range, is derived from the softening temperature and the Fraass breaking temperature. Although each of these parameters indicated a different behaviour with regard to the proportion of impurities and the PE/PP ratio, when put together, we have a very typical behaviour. In each case, technological ageing results in an increase in the plasticity range. Furthermore, an increase in the parameter can be observed for all mixtures regardless of the additive, the proportion of impurities, or the PE/PP ratio.

**Figure 11:**
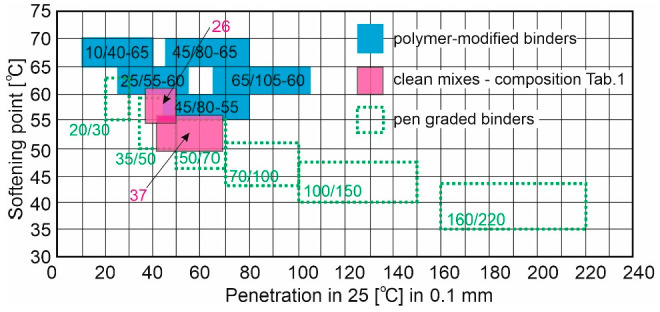
Graphic classification of pen-graded asphalts and modified asphalts as a function of penetration at 25 °C and softening point [36,37].

The results presented in Figure 7 and Figure 8 allow the mixtures analysed to be plotted on a graph showing typical road asphalts and SBS-modified asphalts that are commercially available in Poland, cf. Figure 11.

It can be seen that the mixtures analysed are positioned somewhere between the 35/50 and 50/70 road asphalts and the PMB 25/55-60 and PMB 65/105-60 polymer asphalts, with some overlap with the PMB 45/80-55 asphalt. However, this graph was created on the basis of only two parameters, i.e., penetration and softening temperature. These are not sufficient parameters, even taking into account the previously presented results of breaking temperature and plasticity range tests for comparison to polymer-modified asphalts. This was one of the reasons why it became necessary to determine the rheological parameters of asphalts using different types of rheometers. Therefore, the next subsection contains the results of rheological tests on the mixtures analysed over a wide temperature range.

## 5. Effect of Technological Ageing on Basic Functional Parameters

Rheological studies of mixtures can be carried out over a very wide temperature range, which means that it is possible to characterise asphalt as a material that can be treated as a liquid at elevated (so-called process) temperatures, then at transitional temperatures as a liquid with viscous characteristics or a solid with visco-elastic characteristics, so that at negative temperatures it can be treated as a non-linear-elastic solid. It can be said that, in the case of asphalts, it is reasonable to determine their rheological properties at the process and operational stages of road pavements. Of course, different research methods have been developed over the years in each area. Thus, at elevated temperatures, we use the Brookfield viscosity meter; in the medium temperature range, the DSR dynamic shear rheometer; and at low temperatures, the BBR flexural beam rheometer.

### 5.1. High Temperature Range

**Figure 12:**
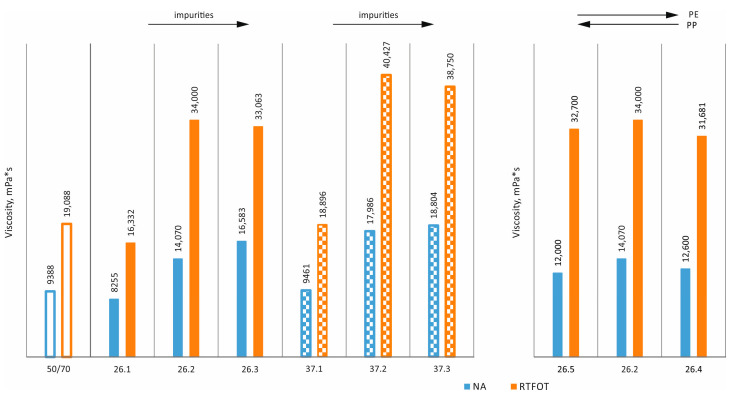
Brookfield viscosity at 90 °C.

**Figure 13:**
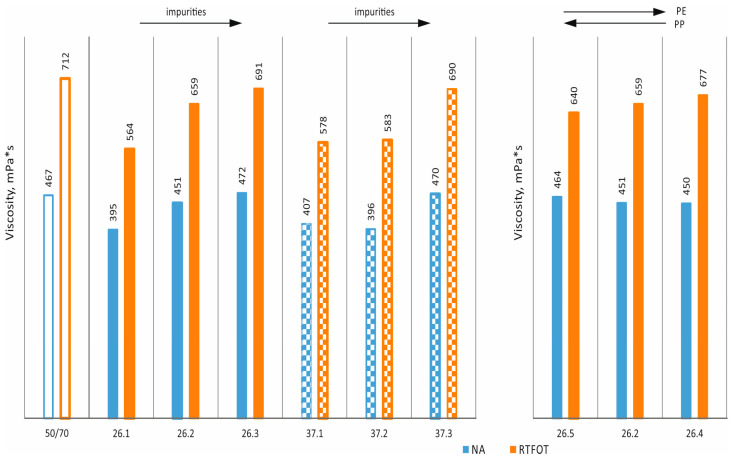
Brookfield viscosity at 135 °C.

Brookfield viscosity tests were performed in accordance with ASTM D4402. The results obtained are shown in Figure 12 and Figure 13. It can be seen that the additives used have little effect on the viscosities obtained at both 90 °C and 135 °C. However, the typical effect of technological ageing, which increases viscosity by about 5 to 10 percent, is clear [36,37].

The use of additives in pure form (without impurities) at 90 °C allows a minimal reduction in viscosity, while an increase in viscosity can be observed as the degree of impurities increases (both in the original state and after RTFOT). By contrast, at a slightly higher temperature of 135 °C, viscosities are at slightly lower or comparable levels.

The level of viscosity and the effect of changes in the determination temperature (thermal sensitivity) translate into values for process temperatures (pumping, production and incorporation temperatures, transport, and end of effective compaction). The Brookfield viscosity results indicate that the use of the analysed additives does not entail significant changes in viscosity, and, therefore, there is no need to modify the temperature of the individual process steps in the production of mineral–asphalt mixtures.

### 5.2. Medium Temperature Range

With regard to tests in the medium temperature range (i.e., below process temperatures but above negative service temperatures) the DSR dynamic shear rheometer [38] is commonly used. It typically conducts tests from about 10 °C to about 70 °C. The DSR rheometer operates in what is known as shear mode and can be controlled either displacementally (predefined, e.g., shear strain) or by torsional moment (predefined, e.g., shear stress). Of course, this makes it possible to carry out studies with any planned loading programme. Nevertheless, over the years, among the tests carried out on asphalts, the most important are the tests of combined shear modulus under strain sinusoidal signal (EN 14770) and the test of repetitive stress–strain creep with two set levels (MSCR—EN 16659) [39]. 

**Figure 14:**
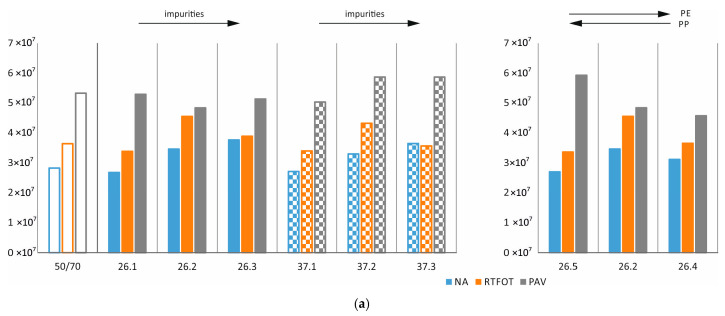

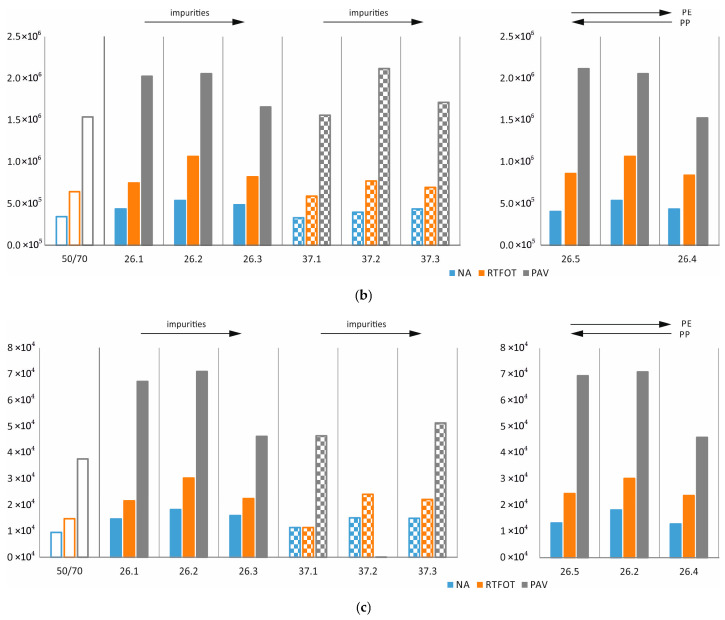
Complex shear modulus norm at (**a**) 10 °C, (**b**) 40 °C, and (**c**) 70 °C—effect of technological and operational ageing (in Pa).

For the first test, the real and imaginary parts of the complex stiffness modulus are determined, as well as the phase shift angle between the given strain pulse and the recorded stress pulse. In this paper, in order to assess the influence of additives, but also of impurities, the PE/PP ratio, and ageing, the presentation of the complex modulus norm was limited to three selected temperatures, i.e., at 10 °C, 40 °C, and 70 °C, cf. Figure 14. By analysing the graphs, it can be seen that ageing causes a significant increase in the value of the shear modulus norm regardless of temperature, type of additive, proportion of impurities, and PE/PP ratio. At 10 °C with additive 26, the increasing proportion of impurities results in a decrease in the modulus norm, while with additive 37, it increases (especially after operational ageing). At 40 °C, it is possible to observe first an increase in the value of the modulus norm at a volumetric share of 20% of the impurities and then a decrease in the value of the modulus norm at a share of 40%. This may indicate that a small proportion of impurities may have a positive effect on the mechanical properties of asphalts with additives, but increasing the amount of impurities leads to a gradual deterioration. Comparing the test results of the base asphalt and the mixtures, it can be seen that in most cases the additives cause an increase in the shear modulus norm. Such an observation does not translate directly into a functional assessment, but it can be assumed that higher values at 70 °C will translate into better deformation resistance of the binder and asphalt mixture. 

From a certain point of view, the MSCR test can replace the so-called elastic recovery test, which is used to evaluate polymer-modified asphalts, cf. Figure 6. The parameters R_0.1 kPa and R_3.2 kPa are calculated as the percentage of reversible (elastic) strain in the total strain recorded in each of the loading cycles with a stress forcing of 0.1 kPa and 3.2 kPa. Higher values mean better results—a higher proportion of the elastic phase. The MSCR test was conducted at 64 °C, at which viscous characteristics dominate over elastic ones.

**Figure 15:**
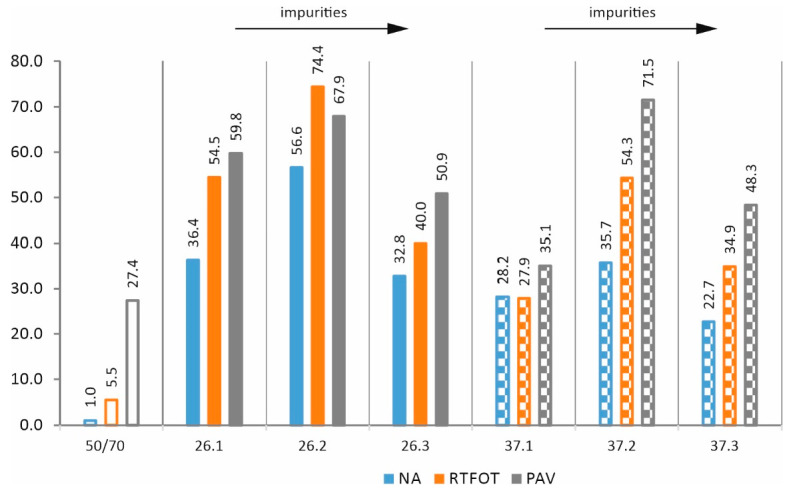
Parameter R_0.1 kPa—elastic recovery at 64 °C (in %).

From Figure 15, it can be seen that additives 26 and 37 have a very positive effect on the values of the R_0.1 kPa parameter. The values for elastic recovery are significantly higher than for the base asphalt. The share of 20% of impurities further enhances this effect, but increasing the amount of impurities to 40% has a negative effect. It can be seen that, as with the norm of complex shearing modulus, there is an optimum value for the proportion of impurities.

**Figure 16:**
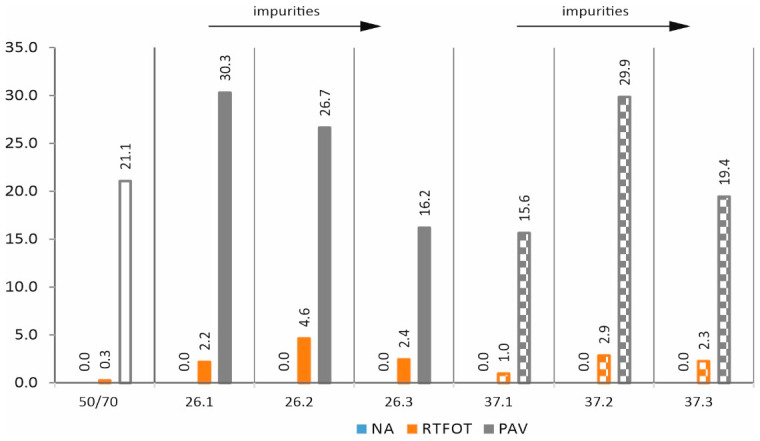
Parameter R_3.2 kPa—elastic recovery at 64 °C (in %).

When the stress pulse has a maximum value of 3.2 kPa (Figure 16), the mixtures in the original state do not show elastic recovery. The situation improves slightly for mixtures after the technological ageing, but significant values for the R_3.2 kPa parameter were only obtained when operational ageing was taken into account. The results after RTFOT ageing in all cases show higher values for the 3.2 kPa parameter for the mixtures than for the base asphalt. After operational ageing, the results are no longer so optimistic, but mixtures 26.1, 26.2, and 37.2 should be distinguished.

### 5.3. Low Temperature Range (Negative Temperature Values, Solid)—BBR Rheometer

**Figure 17:**
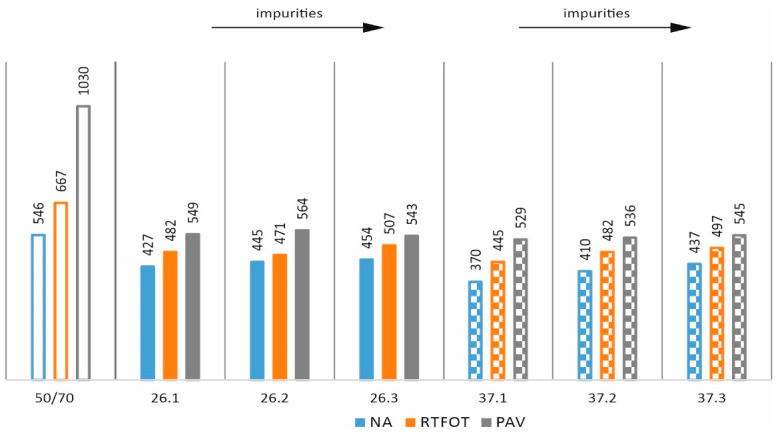
Modulus of stiffness as determined by BBR test [MPa].

The bent beam rheometer (BBR) test was carried out at temperatures of −12 °C, −18 °C, and −24 °C. At all temperatures, a similar effect of ageing and impurities on the results was observed, so only the results obtained at the lowest temperature are presented in this paper. Analysing the results from Figure 17 on the stiffness modulus values, it can be concluded that impurities do not have a major influence on its value and that ageing has a typical and expected effect (i.e., causes the mixture to stiffen). It is worth noting, however, that the subsequent ageing stages affect the base asphalt to the greatest extent, while asphalts modified with additives 26 and 37 age relatively more slowly. Combined with the conclusions from the Fraass breaking temperature test (cf. Figure 9), a positive effect on low-temperature breaking of the mixtures produced on the basis of the mixtures analysed should be expected.

**Figure 18:**
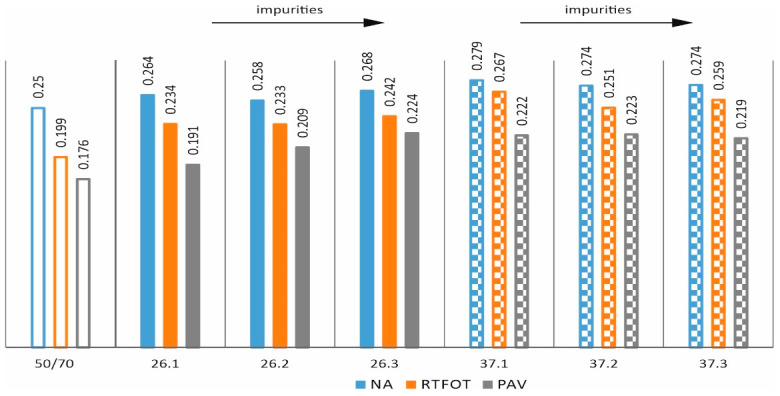
“m” parameter determined by the BBR study [-].

The dimensionless “m” parameter, determined in the BBR test, characterises the slope of the tangent to the stiffness diagram at 60 s of the test as a function of time, i.e., it illustrates the disappearance of viscous characteristics in the tested binder. Evaluating the test results shown in Figure 18, it can be seen that both after the application of pure additives and with an increase in the impurity content, the value of this parameter increases, which is a favourable phenomenon. It can be seen that binders with additives have a steeper tangent slope compared to the base asphalt. On this basis, it can be concluded that the mixtures obtained will be more susceptible than the road comparison binder at a given temperature. The results observed suggest that the use of asphalts with additives can have a positive effect on the low-temperature cracking of mixtures produced from the mixtures analysed.

## 6. Conclusions and Summary

As mentioned earlier, this paper is a continuation of the work [17]. Based on the aforementioned work, two additives, 26 and 37, were selected for detailed analysis. This choice was influenced not only by the position in the ranking developed at [17], but also by the fact that these two additives are produced by pyrolysis from a mixture of plastics, which, in the light of practical applications, can be a perfect improvement in the implementation of the technological process due to the reduced need for segregation. In this paper, a detailed study was carried out on the produced mixtures of selected additives with 50/70 road asphalt, taking into account, among other things, the effect of impurities and the ratio of PE to PP in the additives, as well as technological and operational ageing. An extensive research programme was carried out, performing functional and rheological tests over a wide temperature range. Specific conclusions are presented when discussing the results of the research, while conclusions of a general nature are presented below:

1. The addition of impurities in limited amount not only does not worsen but, in the case of several of the parameters, even improves the properties of the mixtures. Unfortunately, excess impurities lead to deterioration of their properties.
2. When analysing the influence of the mass share of PE and PP, it can be seen that there is certainly an optimum value for the PE/PP ratio. In this study, only three mixtures were tested with values of this ratio of 48/52, 58/42, and 68/32, respectively. The results obtained indicate that the 58/42 ratio is the best; however, this research direction can be taken in further work to identify the optimum PE/PP ratio.
3. Mixtures with additives undergo an ageing process analogous to road asphalts or modified asphalts. The results obtained with the BBR rheometer even show that the degree of ageing, especially operational ageing, is much lower (lower increase in modulus of stiffness) for mixtures with the additives tested than for the base asphalt.
4. Treating the tested mixtures as a market product, it can be concluded that they rank between the 35/50 and 50/70 road asphalts and the PMB 25/55-60 and PMB 65/105-60 polymer asphalts, with some overlap with the PMB 45/80-55 asphalt. Unfortunately, the elastic recoveries obtained (elastic recovery test and MSCR test) are not as high as for asphalts modified with SBS copolymer.
5. Comparing the results of the mixtures and the base asphalt, it can be concluded that, despite the lack of elastic recovery at the polymer asphalt level, features can be identified that are in comparison with the base asphalt. The effect of additives can be particularly beneficial under high-temperature conditions, providing higher resistance to permanent deformation. This was demonstrated for a number of mixture variants by softening temperature tests, shear modulus at 70 °C and MSCR results under post-ageing conditions at 64 °C and a stress of 3.2 kPa. On the other hand, and advantageously, no deterioration of the low-temperature properties was observed in Fraass breaking temperature and BBR rheometer tests, and in some cases, they were even better than for the base asphalt. In addition, basic research has indicated a beneficial increase in the plasticity interval.
6. It is difficult to prejudge whether the presence of oil condensate in the production process (i.e., whether additive 26 or additive 37 is better) has a positive or negative effect on the properties of the resulting mixtures. The results obtained slightly distinguish one or the other additive depending on the study/parameter. On the other hand, it is clear that processing the oil condensate into additives has a positive environmental effect (there is no need to manage the remaining oil after additive production).

## Figures and Tables

**Table 1:** Percentage by weight of plastics in individual additives (PE/PP mass ratio = 58/42).

Additive Number	PE	PP	PET	PS	Oil Condensate
26	57	41	1.5	0.5	none
37	57	41	1.5	0.5	current

**Table 2:** Marking of test samples (base asphalt 50/70).

	26.1	26.2	26.3	26.4	26.5	37.1	37.2	37.3
Percentage share of impurities	0	20	40	20	20	0	20	40
Mass ratio PE/PP	58/42	58/42	58/42	68/32	48/52	58/42	58/42	58/42

## Data Availability

The original contributions presented in the study are included in the article, further inquiries can be directed to the corresponding author.
